# Necking and notch strengthening in metallic glass with symmetric sharp-and-deep notches

**DOI:** 10.1038/srep10797

**Published:** 2015-05-29

**Authors:** Z. D. Sha, Q. X. Pei, Z. S. Liu, Y. W. Zhang, T. J. Wang

**Affiliations:** 1 International Center for Applied Mechanics, State Key Laboratory for Strength and Vibration of Mechanical Structures, Xi’an Jiaotong University, Xi’an 710049, China; 2 Institute of High Performance Computing, A*Star, 138632, Singapore; 3 State Key Laboratory for Strength and Vibration of Mechanical Structures, School of Aerospace Engineering, Xi’an Jiaotong University, Xi’an 710049, China

## Abstract

Notched metallic glasses (MGs) have received much attention recently due to their intriguing mechanical properties compared to their unnotched counterparts, but so far no fundamental understanding of the correlation between failure behavior and notch depth/sharpness exists. Using molecular dynamics simulations, we report necking and large notch strengthening in MGs with symmetric sharp-and-deep notches. Our work reveals that the failure mode and strength of notched MGs are strongly dependent on the notch depth and notch sharpness. By increasing the notch depth and the notch sharpness, we observe a failure mode transition from shear banding to necking, and also a large notch strengthening. This necking is found to be caused by the combined effects of large stress gradient at the notch roots and the impingement and subsequent arrest of shear bands emanating from the notch roots. The present study not only shows the failure mode transition and the large notch strengthening in notched MGs, but also provides significant insights into the deformation and failure mechanisms of notched MGs that may offer new strategies for the design and engineering of MGs.

Metallic glasses (MGs) have received extensive attention as potential structural materials due to their superior mechanical properties such as high strength and large elastic strain^1^,^2^. However, the lack of tensile ductility has been considered the Achilles’ heel of MGs, which significantly limits their structural applications^3^,^4^. The focus of research on MGs, as a consequence, has been on improving their plasticity^5^,^6^,^7^,^8^,^9^,^10^,^11^,^12^. Recent studies suggested that notched MGs can behave quite differently and thus may offer an interesting possibility to tune the properties of MGs^13^,^14^,^15^,^16^,^17^,^18^,^19^,^20^,^21^,^22^,^23^. For example, Zhao *et al*. reported that the plasticity of MGs can be improved to a high value of 10% under compression tests by creating two symmetrical semi-circular notches^21^. Wang *et al*. reported densification and hardening in notched MGs under multiaxial loading^19^. Demetriou *et al*. reported that the fracture toughness in MGs with a single-edge notch is comparable to those of the toughest materials known^13^. Gu *et al*. reported a transition in failure mode from shear banding to cavitation after introducing a single-edge notch in MGs^14^. These studies show that the mechanical properties of notched MGs are intriguing, and clearly, understanding the mechanical properties of notched MGs is of great scientific and technological interest.

Despite the fact that notched MGs offer a suite of fascinating mechanical properties^13^,^14^,^16^,^17^,^19^,^20^,^21^,^22^,^23^, a fundamental understanding of the correlation between their failure behavior and notch depth/sharpness is still lacking. Those previous works^13^,^14^,^15^,^16^,^17^,^18^,^19^,^20^,^21^,^22^,^23^ all prompt an important question: How does the notch depth/sharpness affect the deformation and failure behavior of MGs? In the present work, we address this question by conducting molecular dynamics (MD) simulations on symmetrically notched MGs with variation of notch depth and notch sharpness under tensile loading. Our work unambiguously demonstrates that the failure mode and strength of notched MGs depend on not only the notch depth but also the notch sharpness. It is found that the stress gradient becomes increasingly larger with increasing notch depth. In addition, for MGs with sharp notches, the decrease of stress concentration factor is found to be slower than that with blunt notches. With increasing both notch depth and sharpness, surprisingly, the failure mode shifts from shear banding to necking. Accompanying this transition, a large notch strengthening is observed. Our atomistic simulations provide significant insights into the deformation and failure mechanisms of notched MGs.

## Results

**Figure 1:**
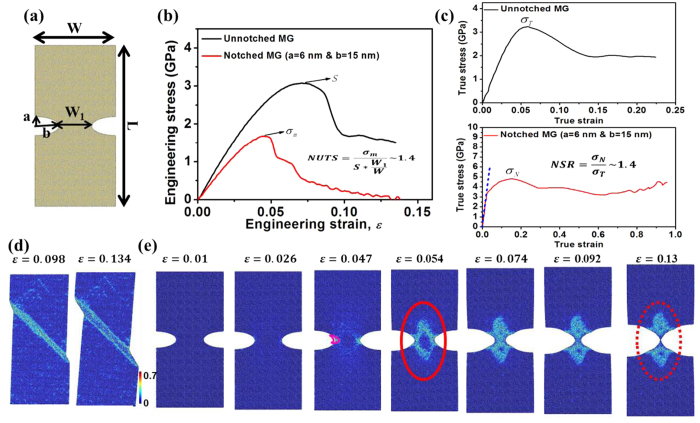
Necking and notch strengthening in a MG with symmetric sharp-and-deep notches. (**a**) Notched MG structure investigated, *W* = 50 nm, *L* = 100 nm, *W*_*1*_ = 20 nm, *a* = 6 nm, and *b* = 15 nm. (**b**) The engineering stress–strain curves for unnotched and notched MGs. The engineering stress is defined as F(t)/(W*T). The engineering strain (*ε*) is defined as (*L(t)-L)/L*. (**c**) The true stress-strain curves for unnotched and notched MGs, respectively. For unnotched MG, F(t)/(W(t)*T(t)) and (*WT-W(t)T(t))/WT* are defined as the true stress and true strain, respectively. For notched MG, *F(t)/(W*_*1*_(t)*T(t)) and (*W*_*1*_*T-W*_*1*_*(t)T(t))/W*_*1*_*T* are defined as the true stress and true strain, respectively. The dotted blue line indicates the linear elastic regime. (**d**-**e**) A sequence of snapshots show the deformation and failure mechanisms for unnotched and notched MGs, respectively. The color in (**d**) and (**e**) indicates the atomic local shear strain. The pink circle in (**e**) outlines the area of the plastic zone at the notch root. The red circle in (**e**) indicates the V-shaped shear regions. The dotted red circle in (**e**) indicates the necking behavior.

Figure 1(a) shows the atomic configuration of a MG with symmetric sharp-and-deep notches, in which *a* and *b* represent the minor and major axis of the elliptic notch, respectively. *W* and *L* are the initial width and length of the MG specimen, respectively. The initial width of the reduced cross-section in the notch region is *W*_*1*_. In order to compare the *overall* stress–strain curves for both notched and unnotched MGs, we define the engineering stress as F(t)/(W*T), where *F(t)* is the load, *T* is the initial thickness of the MG specimen, and *t* is the loading time. The engineering strain is defined as (*L(t)-L)/L*. Figure 1(b) shows the comparison of the engineering stress vs. engineering strain curves between the unnotched sample with *W* = 50 nm and *L* = 100 nm, and the sample of the same size but symmetrically notched with *a* = 6 nm and *b* = 15 nm. It is seen from Fig. 1(b) that the ultimate tensile strength (UTS) of the notched MG is lower than that of the unnotched MG. The normalized ultimate tensile strength (NUTS) is used to characterize the notch sensitivity of the failure strength^18^,^24^,^25^. Here, NUTS is defined as 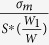, where *σ*_*m*_ is the UTS of the notched MG from MD simulation, and *S* is the UTS of the unnotched MG. Note that NUTS >1 is defined as notch strengthening while NUTS < 1 as notch softening^18^,^25^. In this case, the symmetric notches reduce the cross-sectional area of the MG specimen by 60%, but only reduce the strength by 44%. As a result, NUTS is as large as 1.4, showing a large notch strengthening.

To better show this large notch strengthening behavior, the true stress-strain curves for the unnotched and notched MGs are also plotted in Fig. 1(c). For the unnotched MG, the true stress is defined as F(t)/(W(t)*T(t)), where *W(t)* and *T(t)* are the width and thickness of the unnotched MG during deformation. The true strain is defined as (*WT-W(t)T(t))/WT*. For the notched MG, the real width of the reduced cross-section in the notch region during deformation (*W*_*1*_*(t)*) should be used. As a result, *F(t)/(W*_*1*_(t)*T(t)) and (*W*_*1*_*T-W*_*1*_*(t)T(t))/W*_*1*_*T* are defined as the true stress and true strain, respectively. As different widths are used in the definitions of true strain, we separately plot the true stress-strain curves for the notched and unnotched MGs in Fig. 1(c). To characterize the notch strengthening effect, one can use the notch strength ratio (NSR)^16^, which is defined as 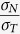, where *σ*_T_ and *σ*_N_ are the maximum strength of the unnotched and notched MG, respectively. We find that NSR is about 1.4, thus consistent with the value of NUTS, further supporting the notch strengthening.

The dotted blue line shown in the lower panel of Fig. 1(c) indicates the linear elastic stage up to a true strain of ~2.0%, in which the true stress for the notched MG has reached the maximum strength of the unnotched MG (*σ*_T_) due to the stress concentration at the notch root. With further loading, plastic deformation begins for shear bands (SBs) initiation/propagation. There is an apparent strain-hardening stage till the reach of *σ*_N_ at the true strain of ~15%, which is much larger than the true strain of ~5.8% corresponding to the unnotched strength *σ*_T_. Clearly, the apparent strain-hardening arising from the introduction of notches contributes to the enhancement in the true failure strength, leading to the observed notch strengthening.

Figure 1(d-e) show a sequence of snapshots to reveal the deformation and failure mechanisms of unnotched and notched MGs, respectively. For the unnotched MG, a SB forms and subsequently propagates rapidly across the sample, leading to a sudden and rapid stress drop as shown in Fig. 1(b-c)^26^. It should be noted that the plastic flow after the SB formation corresponds to a stable sliding of the SB as shown at the engineering strain of 13.4% in Fig. 1(d)^18^,^26^,^27^,^28^. For the notched MG as shown in Fig. 1(e), there is no obvious structural change (i.e., plastic deformation) before the engineering strain of 2.6% (corresponding to the true strain of 2.0%). With further loading, plastic deformation occurs at the notch roots due to the stress concentration around notches. At the engineering strain of 4.7% (corresponding to the true strain of 15%), the diameter of the plastic zone at the notch root outlined by the pink circle in Fig. 1(e) is estimated to be ~5.35 nm, which is comparable with the SB width^14^. Then the SBs start to initiate from each notch root. During the propagation of SBs, however, two SBs intersect and form V-shaped shear regions as shown in Fig. 1(e) at the engineering strain of 5.4%. This interaction of SBs arrests their further propagation^20^,^21^,^22^. Thereafter, necking occurs at the notch region as shown at the engineering strain of 13% in Fig. 1(e). It should be emphasized that our study is a first direct atomistic simulation of the transition from shear banding to necking in notched MGs.

**Figure 2:**
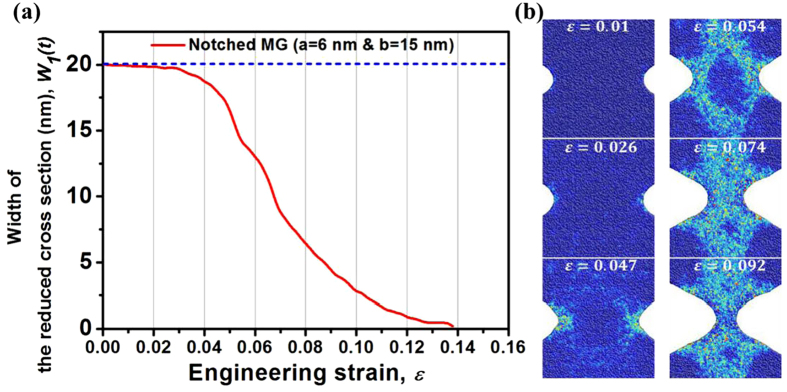
(a) The change of width of the reduced cross-section in the notch region (*W*_*1*_*(t)*) during deformation. (**b**) Several close-up views of the notch root region at different stages of loading. The color in (**b**) indicates the atomic local shear strain.

In the above, we have demonstrated the necking and notch strengthening in a MG sample with symmetric sharp-and-deep notches. To further understand the physical origin of these interesting observations, Fig. 2(a) shows the trend of width of the reduced cross-section in the notch region (*W*_*1*_*(t)*) during deformation. In order to show the change more clearly, Fig. 2(b) shows several close-up views of the notch root region at different stages of loading. At the initial stage, the change of *W*_*1*_ is insignificant. But *W*_*1*_ starts to drop rapidly after the engineering strain of ~2.6%, which corresponds to the end of the linear elastic stage at the true strain of ~2.0% and the true stress has reached the σ_*T*_. During the subsequent apparent strain-hardening, SBs initiation and arrest, and necking as shown in Fig. 2(b), *W*_*1*_ continues to drop monotonically as shown in Fig. 2(a).

In previous experimental works on notched MGs, Qu *et al*.^12^ reported a small NSR of 1.14; Zhao *et al*.^20^,^21^,^22^ reported the interaction of SBs and the formation of the V-shaped shear region; and Wang *et al*.^19^ observed the contraction along the notch region. These observations are all observed in our present simulations. But these previous works did not report necking and large notch strengthening. We believe that this is due to the high sensitivity of the failure modes to the notch depth and notch sharpness. Below, we examine in details the effects of notch depth and notch sharpness on the deformation and failure behavior of MGs.

**Figure 3:**
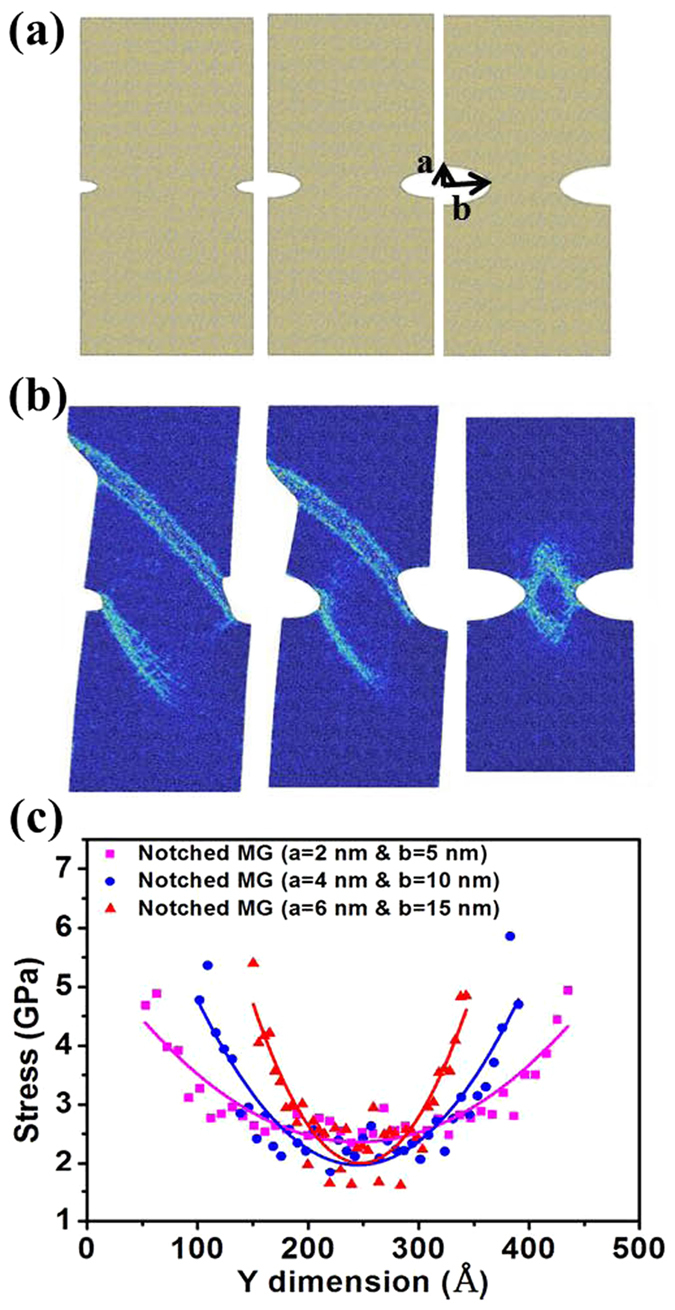
Illustrations of the effect of notch depth. (**a**) Notched MG structures investigated. Left panel: *a* = 2 nm and *b* = 5 nm, Middle panel: *a* = 4 nm and *b* = 10 nm, and Right panel: *a* = 6 nm and *b* = 15 nm, the same notch size in Fig. 1. (b) Snapshots show the failure behaviors for the notched MGs with the different notch depths. The color in (**b**) indicates the atomic local shear strain. (**c**) The stress profile along the reduced cross-section in the notch region with three different notch depths.

Figure 3 illustrates the effect of notch depth. The atomic configurations of MGs are of the same sample sizes, but with symmetric elliptic notches of various aspect ratios as shown in Fig. 3(a). For the left panel, *a* = 2 nm and *b* = 5 nm; for the middle panel, *a* = 4 nm and *b* = 10nm; and for the right panel, *a* = 6 nm and *b* = 15 nm. Figure 3 (b) shows that their failure modes shift from shear banding (the left and middle panels) to necking (the right panel). Interestingly, for the left and middle panels, there is only one SB being nucleated and subsequently propagating from each notch root. For the right panel, however, two symmetrical SBs are nucleated and subsequently propagate from each notch root. As a result, the SBs impinge and form intersections. Importantly, such interactions cause the arrest of the SBs propagation. To further examine the physical origin of this transition in failure mode, Fig. 3(c) plots the stress profile along the reduced cross-section in the notch region. It is found that the stress gradient becomes increasingly larger with increasing notch depth. It is known that a large stress gradient can cause SBs to initiate with ease but to propagate with difficulty^20^,^21^,^22^. It is seen from Fig. 3(c) that the two deepest notches cause the largest stress gradient. Consequently, due to the combined effects of large stress gradient and the impingement of SBs, necking takes place at the notch region with further loading.

**Figure 4:**
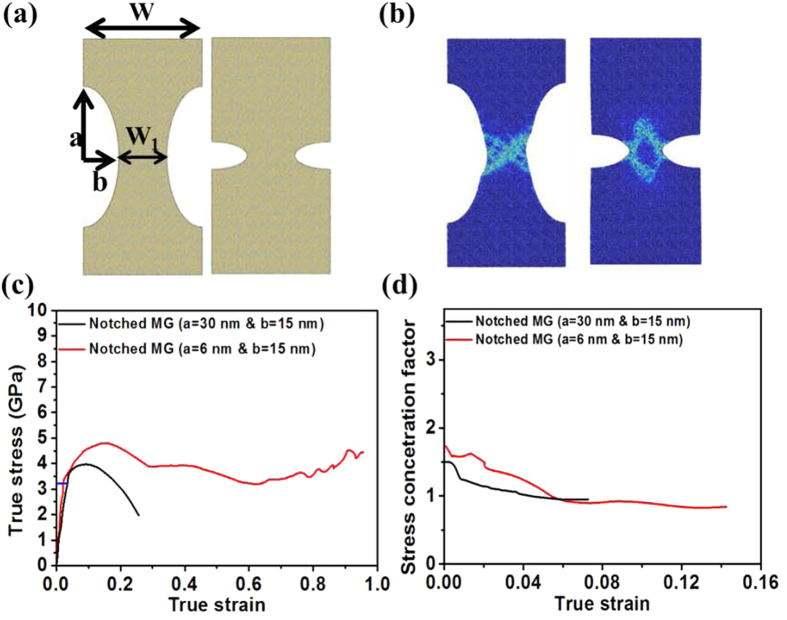
Illustrations of the effect of notch sharpness. (**a**) Notched MG structures investigated. Left Panel: *a* = 30 nm and *b* = 15 nm, and Right panel: *a* = 6 nm and *b* = 15 nm, the same notch size in Fig. 1. (b) Snapshots show the failure behaviors for the notched MGs with blunt and sharp notches, respectively. The color in (**b**) indicates the atomic local shear strain. (**c**) The true stress–strain curves for MGs with sharp and blunt notches, respectively. The blue line indicates the maximum strength of unnotched MG ***σ***_*T*_. (**d**) The trend of stress concentration factor at the notch root during deformation up to the onset of the SB formation. The stress concentration factor is defined as the ratio of the stress at the notch root over the stress on the reduced cross-section.

The effect of notch sharpness is illustrated in Fig. 4. Figure 4 (a) illustrates two MG samples of the same notch depth *b* = 15 nm, but different root radii with *a* = 30 nm for the left panel (blunt notch) and *a* = 6 nm for the right panel (sharp notch). The snapshots in Fig. 4(b) show that the failure mode for the MG with blunt notches is shear banding, which is quite different from that with sharp notches. Their corresponding true stress-strain curves are shown in Fig. 4(c). The difference in the initial stage of stress-strain curves is insignificant. However, the MG with sharp notches has a more obvious strain-hardening stage till the true strain of ~15%. As a result, the maximum strength of the MG with sharp notches is higher than that of the MG with blunt notches. Figure 4(d) shows the trend of stress concentration factor at the notch root during deformation up to the onset of the SB formation. We define the stress concentration factor as the ratio of the stress at the notch root over the stress on the reduced cross-section^21^,^29^. The stress concentration factor in the MG with sharp notches is higher than that with blunt notches. Therefore, it is seen from Fig. 4 (c) that at the end of the linear elastic stage around the true strain of 2.0%, the true stress in the MG with sharp notches reaches the maximum strength of unnotched MG *σ*_T_ (indicated by the blue line in Fig. 4 (c)), while the true strain of 3.7% beyond the linear elastic stage is needed for the MG with blunt notches to reach *σ*_T_. Hence more plastic deformation is required in the MG with sharp notches in order to trigger SB initiation/propagation. On the other hand, the decrease of stress concentration factor in the MG with sharp notches is slower than that with blunt notches. As a result, the apparent strain-hardening stage in the MG with sharp notches is larger than that with blunt notches. Therefore, these combined effects of larger stress concentration factor and its slower decrease may explain why the MG sample with sharp notches has a more obvious strain-hardening stage and the according higher maximum strength than that with blunt notches.

## Discussion

It is instructive to discuss the effects of loading temperature and strain rate applied in our MD simulations. At room temperature, MGs fail by localized shear banding, which is referred to inhomogeneous deformation. At higher temperatures, it has been found that MGs can deform homogeneously^30^. In the present work, a low loading temperature of 50 K is used in order to capture the inhomogeneous deformation (shear failure) of MGs^26^. Another reason for using the low loading temperature is to reduce the thermal fluctuation in MD simulations to facilitate the atomic investigation of deformation. As for the strain rate used in the MD simulations, it is higher than that used in experiment mainly due to the limitations of computational method. However, the MD simulation with such high strain rate can still capture SB initiation, formation, and propagation. For these reasons, we focus on major mechanistic features and trends in behavior when interpreting MD simulation results^14^.

It should be emphasized that there has been much experimental and simulation work on the small-scale MGs^6^,^7^,^14^,^31^,^32^. For example, Guo *et al*. reported the tensile ductility and necking for MGs with dimensions of about 100 nm^32^. Transition from a strong-yet-brittle to a stronger-and-ductile state was also reported through size reduction of MGs^6^,^7^,^31^. Gu *et al*. investigated the deformation and failure mechanisms in nanoscale MGs^14^. In the present work, we focus on the correlation between failure mode and notch geometries of small-scale MGs.

In summary, we have performed MD simulations to investigate the effects of notch depth and notch sharpness on the deformation and failure behavior of MGs. A transition from shear banding to necking and large notch strengthening are observed for a MG sample with symmetric sharp-and-deep notches. Deep and sharp are the two requirements for symmetric notched MGs to exhibit such necking and large notch strengthening. Our results find that failure mode and strength in notched MGs depend critically on the notch depth and notch sharpness. By increasing the notch depth and the notch sharpness, we observe a failure mode transition from shear banding to necking, also a large notch strengthening. These results provide useful insights into fundamental failure mechanisms of notched MGs that may help shape strategies for design and engineering of MGs.

## Methods

We perform MD simulations using the Large-scale Atomic/Molecular Massively Parallel Simulator (LAMMPS)^33^. The Cu_50_Zr_50_ MG contains ~1.7 million atoms in a thin film geometry of dimensions of 100.0 (x) × 50.0 (y) × 6.23 (z) nm^3^. A constant integration time step of 2 fs is used in all the simulations. The atomic interactions within the Cu−Zr MGs are modeled using the embedded atom method (EAM) potential^34^,^35^. In constructing the sample, a small cube (~13000 atoms) with periodic boundary conditions (PBCs) along all three dimensions is first equilibrated at 2000 K for 2 ns, and then cooled at a quenching rate of 10^9^ K/s to 50 K, at zero external pressure. The simulation samples are then constructed by replications of the small cube, then annealed for 0.5 ns at 800 K, and finally brought back to 50 K^26^,^27^,^28^. For uniaxial tensile tests, PBCs are imposed along the x- and z-directions while free surface is used along the y-direction. An engineering strain rate of 10^8^ s^−1^ along the x-direction is used at a low temperature (50 K). The stress is calculated from the normal tensor component of the Virial stress along the loading direction^26^. Symmetric superficial elliptic notches with various aspect ratios are used to investigate the effects of notch depth and shape on the deformation and failure behaviors of MGs.

## Additional Information

**How to cite this article**: Sha, Z. D. *et al*. Necking and notch strengthening in metallic glass with symmetric sharp-and-deep notches. *Sci. Rep*. **5**, 10797; doi: 10.1038/srep10797 (2015).
